# Short term plasticity within the basal ganglia - a systems level computational investigation

**DOI:** 10.1186/1471-2202-12-S1-P145

**Published:** 2011-07-18

**Authors:** Mikael Lindahl, Jeanette Hellgren Kotaleski

**Affiliations:** 1 Dept. of Computational biology, School of Computer Science and Communication, Royal Institute of Technology, AlbaNova University Center, Stockholm, SE-106 91, Sweden; 2 Stockholm Brain Institute, Karolinska Institute, SE-171 77 Stockholm, Sweden

## 

Striatal direct pathway medium spiny neurons (MSNs) converge, with inhibitory synapses onto output nuclei substantia nigra reticulata (SNr), which keep neurons in the thalamus, superior colliculus and pendunculopontine nuclei under tonic inhibition [1]. Recent experimental findings [2] have found short term facilitation in MSN synapses onto SNr neurons. We investigate the functional consequences of these findings using a basal ganglia system level model, with spiking MSNs modeled according to Izhikevich’s simple model [3] and with facilitating synapses [4] fitted to data in [2]. The model is implemented in the NEST [5] simulator. We quantify how striatal populations of MSNs can control activity in SNr neurons, and to what extent this depends on having weak static, strong static and facilitating synapses between MSNs and SNr neurons.

Our simulation experiments predict that facilitating synapses allow baseline firing of presynaptic MSNs without suppressing target SNr neurons, while burst activation of only a few of these presynaptic striatal neurons can suppress the activity of one SNr neuron. This is in accordance with extracellular recordings in awake animals [6], where task dependent activity is transferred from a broad striatal population to a smaller subpopulation, responding increasingly stronger during learning of a task dependent behavior.
